# Transgenerational Effects of Cadmium and Copper Exposure on Development, Reproduction, and Midgut Integrity in *Culex pipiens* (Diptera: Culicidae): Implications for Vector Ecology Under Metal Pollution

**DOI:** 10.3390/biology14081004

**Published:** 2025-08-05

**Authors:** Ahmed I. Hasaballah, Ramy E. El-Ansary, Mahmoud M. Zidan, Areej A. Al-Khalaf, Abdelwahab Khalil

**Affiliations:** 1 Zoology and Entomology Department, Faculty of Science, Al-Azhar University, Cairo 11651, Egypt; 2 Biology Department, College of Science, Princess Nourah bint Abdulrahman University, Riyadh 11671, Saudi Arabia; aaalkhalaf@pnu.edu.sa; 3 Entomology Division, Zoology Department, Faculty of Science, Beni-Suef University, Beni-Suef 62521, Egypt

**Keywords:** *Culex pipiens*, cadmium, copper, transgenerational toxicity, fecundity, histopathology, aquatic pollution, bioindicator

## Abstract

Heavy metal contamination in freshwater ecosystems poses persistent threats to aquatic organisms and public health. This study evaluates the transgenerational toxicity of cadmium chloride and copper sulfate on *Culex pipiens*, focusing on development, reproduction, and midgut histopathology over two successive generations. Larval bioassays showed cadmium chloride to be more toxic than copper sulfate, with early instars exhibiting higher sensitivity (LC_50_ = 8.66 μg/L for Cd; 175.63 μg/L for Cu). Both metals significantly delayed larval and pupal development, reduced fecundity, and decreased egg hatchability in a dose-dependent manner. Histopathological examination revealed midgut epithelial degeneration, vacuolation, and brush border loss, with copper sulfate inducing more severe cytotoxicity. These findings confirm that sublethal, chronic metal exposure can impair physiological and reproductive traits across generations. Moreover, this study highlights the utility of mosquitoes as sensitive bioindicators of aquatic pollution, and underscores the long-term ecological implications of heavy metal contamination on vector dynamics and disease transmission.

## Simple Summary

Heavy metal pollution in freshwater environments can harm mosquito populations, which are important both ecologically and as disease vectors. This study examines the impact of long-term exposure to cadmium and copper on *Culex pipiens*, a common mosquito species, across two generations. We found that cadmium was more toxic than copper, especially to early-stage larvae. Both metals delayed development, reduced egg-laying, and lowered the hatching success of eggs. Furthermore, the internal tissues of mosquito larvae—specifically the midgut—showed clear signs of damage, such as cell degeneration and structural breakdown, especially after copper exposure. These negative effects were observed not only in the directly exposed mosquitoes, but also in their offspring. This suggests that chronic metal exposure can have lasting biological impacts across generations. Our findings highlight the use of mosquitoes as sensitive indicators of water pollution and raise concerns about how such pollutants might influence mosquito populations and the spread of diseases they carry.

## 1. Background

Heavy metal pollution in aquatic habitats is a global environmental concern, with both ecological and public health implications. These metals, including cadmium and copper, are introduced into freshwater ecosystems through industrial discharge, urban runoff, and agricultural effluents. Once released, they persist in the environment and can accumulate in aquatic organisms, disrupting physiological functions and altering ecosystem dynamics [1,2].

Among aquatic invertebrates, insects such as mosquitoes are particularly vulnerable to heavy metal contamination. Their aquatic larval stages make them ideal candidates for biomonitoring, given their high sensitivity to pollutants, short life cycles, and ecological relevance [3]. Previous studies have shown that metal exposure can impair insect growth, development, survival, and reproduction [4,5], and may even lead to adaptive responses such as increased tolerance or resistance to insecticides [6,7].

Cadmium and copper, two commonly encountered metals in polluted water bodies, exert both lethal and sublethal effects on aquatic insects. Cadmium, a non-essential metal, is highly toxic even at low concentrations, interfering with enzymatic activities and disrupting endocrine function [8]. Copper, although essential in trace amounts, can also be toxic when accumulated beyond physiological limits, leading to oxidative stress and tissue damage [9,10].

*Culex pipiens*, a widespread mosquito species and important vector of various arboviruses, frequently breeds in stagnant, metal-contaminated water sources. Understanding how heavy metals affect its life history and physiological traits is critical for predicting population trends and disease risk under polluted conditions. Most prior studies have focused on single-generation responses and external phenotypic traits such as mortality or fecundity [11,12]. However, there is a lack of data regarding transgenerational effects and internal tissue-level impacts, such as histopathological damage.

Therefore, this study aims to evaluate the larvicidal activity of cadmium chloride and copper sulfate against *Culex pipiens*, assess their effects on developmental timing, fecundity, and egg hatchability across two successive generations, and investigate histopathological alterations in larval midgut tissues following sublethal exposure.

We hypothesize that chronic exposure to sublethal concentrations of cadmium and copper induces cumulative physiological and histological impairments that manifest across generations in *Culex pipiens*. This study uniquely integrates transgenerational life history analysis with histomorphometric midgut assessments to reveal potential long-term consequences of metal pollution on mosquito biology and ecology.

## 2. Materials and Methods

### 2.1. Mosquito Colony

Larvae of *Culex pipiens* were generously obtained from the colony established at the Department of Zoology, Faculty of Science, Tanta University, Egypt, and reared until the next generation to obtain first instar larvae for further exposure. For two-generations assays, the domesticated strain is better than the field strain, which may not reach the 2nd generation in the laboratory. Larvae were raised in 40 cm white enamel bowls with a capacity of 1000 mL containing dechlorinated water, at 27 ± 2 °C, 75 ± 5% relative humidity, and a 14–10 h day/night photoperiod. Larvae were supplied ad libitum with fish food as a diet. The produced pupae were transferred into cups filled with dechlorinated water and maintained in (30 × 30 × 30 cm) wooden cages until emergence of adults. A sponge piece dipped in a sugar solution (10%) was provided in each cage. For reproduction and development purposes, 3- to 4-day old females were allowed to feed on pigeon blood and lay egg rafts on cups filled with dechlorinated water provided in each cage.

### 2.2. Chemicals

Cadmium chloride (CdCl_2_) (Sigma-Aldrich, Rockville, MD, USA) and copper sulfate (CuSo_4_) (Sigma-Aldrich, USA) were used for preparation of stock solutions of Cd and Cu, respectively. A stock solution of 1000 μg/L was prepared for both metals. From these solutions, four test concentrations of (1, 2, 4, and 8 μg/L) and (125, 250, 500, and 1000 μg/L) were prepared for cadmium and copper, respectively, in addition to controls. Selected concentrations were below the human safe limit for cadmium [13] and copper [14] in fresh water.

### 2.3. Larvicidal Activity

Larval development was assessed for *Culex pipiens* after exposure to metal pollutants according to the World Health Organization protocol [15]. Briefly, 25 larvae, less than one day after emergence, were exposed to different toxicant concentrations of the relevant metal salts. Larvae were treated with different concentrations mentioned previously, and each concentration was replicated five times. Each replicate was maintained in a plastic cup made of polyethylene containing 25 larvae/250 mL of the relevant solution. Larvae in the control groups were reared in dechlorinated water (without toxicant). The same amount of larval diet was supplied for all treatments. Larvae were observed until pupation, and dead organisms were removed. Larval mortality was recorded daily to evaluate lethal concentrations in each larval instar in addition to the pupal stage.

### 2.4. Development Time of the Immature Stages

Twenty-five newly hatched larvae were treated with the previously determined concentrations, alongside the untreated, to investigate their impact on the development of the mosquito larvae in the laboratory. Larval development time—calculated as the amount of time a larval instar needs to reach the next instar of all larval instars (L_1_–L_4_)—was determined. The larvae were provided with a daily larval diet. Pupal development time, estimated as the period between entering the pupal stage and adult emergence, was also determined. For each treatment, five replicates were tested. Observations were continued until all larvae/pupae in all treatments died or emerged as adults. Later, emerged adults from all replicates within treatments were merged and kept in separate cages to continue with the second-generation larvicidal assay.

### 2.5. Fecundity and Egg Hatchability

In both investigated generations, the same ratio of males to females that survived and emerged from each concentration were collected and allowed to mate normally in standard cages alongside the control to investigate the effect of accumulated heavy metals on female fecundity and egg hatchability. Fecundity was recorded and calculated for females that successfully deposited their eggs, as described by Rak and Ishii [16]. Briefly, deposited eggs were meticulously gathered from cages 3–4 days post-feeding. Fecundity was then determined by summing the number of eggs divided by number of females mated and survived till the end of the experiment. The egg hatchability (%) was calculated according to the following equation:

$$Egg-hatchability\;\%=\frac{A}{B}\;\times \;100$$

where (A) is the number of hatched eggs, and (B) is the number of laid eggs.

### 2.6. Histopathological Examination and Histomorphometry Measurements

Less than a day after they emerged, the newly emerged larvae were exposed to the LC_50_ concentration of CdCl_2_ and CuSO_4_; then, the 3rd instar larvae from both tested generations were picked up for further investigations. Along with the control, the 3rd instar treated larvae were washed with 0.9% saline solution and then fixed with 10% formalin solution. Paraffin wax was used to implant the larvae, and the melted blocks were left for 3 h at 25 ± 2 °C to cool. Thereafter, blocks were sectioned transversely (3 μm) using a microtome Leica-cryocut-1800 (Leica Biosystems, Deer Park, IL, USA), and staining was done on mounted slides using eosin and haematoxylin. Light microscopy was used to examine morphological changes using built-in LAS EZ software V 4.13 with a Leica DM750 compound microscope supplied with a LEIKA ICC50 W camera (Leica Biosystems, Deer Park, IL, USA).

To measure several morphological characteristics of the midgut, such as epithelial thickness, the degree of cellular deterioration, and any structural abnormalities, we used image analysis software. We assessed potential alterations in the midgut’s architectural composition caused by chemical exposure. The data gathered over several generations were analyzed to determine the long-term consequences of chemical exposure. The means (M) and standard error (S.E.) of each set of data were determined [17,18]. After establishing the test’s significance (*p*-value) at the 5% or 1% level, ANOVA was utilized to assess the interaction effects in more detail. Throughout the entire statistical analysis, the program SPSS for Windows, version 10.0, was utilized.

### 2.7. Statistical Analysis

Prior to statistical analysis, data were assessed for compliance with parametric assumptions. Development time and fecundity data were transformed using Box-Cox transformation to achieve normality and homoscedasticity. Egg hatchability proportions were arcsine square-root transformed to stabilize variance. Homogeneity of variance was evaluated using Levene’s test and the F-test for equality of variances, while normality was assessed via the Shapiro–Wilk test. Mortality data from larvicidal bioassays were analyzed using probit regression to determine lethal concentrations (LC_50_ and LC_90_) with 95% confidence intervals. Probit models were fitted to concentration–mortality relationships for each developmental stage and generation, with model adequacy assessed through Chi-squared goodness-of-fit tests. A three-way analysis of variance (ANOVA) was conducted to examine the main effects and interactions of heavy metal type (cadmium chloride vs. copper sulfate), developmental stage (larval vs. pupal), and generation (F_1_ vs. F_2_) on development time. Transgenerational effects of metal exposure on reproductive parameters were evaluated using two-way ANOVA, with metal concentration and generation as fixed factors. Fecundity and egg hatchability served as dependent variables in separate analyses. To control for family-wise error rates associated with multiple comparisons, Bonferroni corrections were applied to all pairwise comparisons in both three-way and two-way ANOVA models. Effect sizes were calculated using partial Eta-squared (η^2^ₚ) to assess the practical significance of observed differences. All statistical analyses were performed using IBM SPSS Statistics version 27.0 (IBM Corporation, Armonk, NY, USA). Statistical significance was determined at α = 0.05 for all tests, with Bonferroni-adjusted *p*-values reported where applicable.

## 3. Results

### 3.1. Metal Toxicity

In this work, different concentrations of cadmium chloride and copper sulfate were evaluated against 1st to 4th larval instars and pupae of *Culex pipiens* in two successive generations (Table 1 and Table 2). The calculated LC_50_ values revealed that cadmium chloride was more toxic to early instars than to later ones. In the first generation, the 1st instar larvae exhibited the highest susceptibility, with an LC_50_ of 8.66 μg/L, compared to the 4th instar, which had a significantly higher LC_50_ of 20.8 μg/L. A similar trend was observed in the second generation, where LC_50_ values were 6.84 μg/L for the 1st instar and 18.85 μg/L for the 4th instar, indicating that earlier stages are consistently more sensitive to cadmium exposure.

Regarding copper sulfate, a parallel pattern was observed. In the first generation, the LC_50_ for the 1st instar was 175.63 μg/L, compared to 345.58 μg/L for the 4th instar. In the second generation, the 1st instar again showed greater sensitivity (LC_50_ = 139.22 μg/L) than the 4th instar (LC_50_ = 302.56 μg/L). These results demonstrate that early larval stages of *Culex pipiens* are more vulnerable to both metals, and that toxicity tends to decrease with larval development. Furthermore, a slight increase in LC_50_ values from the first to second generation may suggest developing tolerance or physiological acclimation. These findings underscore the importance of LC_50_-based comparisons for accurately assessing stage- and generation-specific susceptibility to toxicants.

It is obvious from Table 1 and Table 2 that the pupal stage was more resistant to metal concentrations than all mosquito larval instars in both generations.

### 3.2. Development Time of the Immature Stages

**Figure 1:**
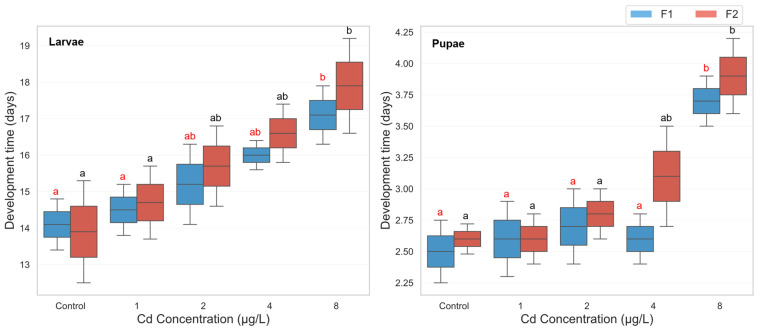
Larval and pupal development time in two successive generations of *Culex pipiens* following long-term exposure to varying concentrations of cadmium chloride. Significant differences (*p* < 0.05, three-way ANOVA with Bonferroni test) in the developmental period are indicated by different red letters for the first generation and different black letters for the second generation.

The three-way ANOVA revealed a significant main effect of cadmium concentration on development time (F(4, 39) = 13.351, *p* < 0.001, partial η^2^ = 0.578), indicating that increasing cadmium exposure significantly delayed mosquito development. Likewise, a strong main effect of developmental phase was observed (F(1, 39) = 202.390, *p* < 0.001, partial η^2^ = 0.838), with larvae requiring significantly more time to develop than pupae across all treatments. Although the effect of generation approached significance (F(1, 39) = 3.677, *p* = 0.063, partial η^2^ = 0.086), no statistically significant interactions were detected among the three factors, including concentration × phase (*p* = 0.795), concentration × generation (*p* = 0.807), phase × generation (*p* = 0.636), or the three-way interaction (*p* = 0.978). This suggests that the effect of cadmium on development time was largely consistent across generations and life stages. Descriptive statistics confirmed a progressive increase in both larval and pupal development durations with rising cadmium concentrations across generations. Larval development time increased from an average of 14.0 days in the control group to 17.5 days at 8 µg/L, while pupal development increased from 2.55 to 3.8 days (Figure 1). Post hoc pairwise comparisons (Bonferroni-adjusted) showed that larval development in both generations was significantly prolonged at the highest cadmium concentration (8 µg/L) compared to the control. Specifically, in the F1 generation, the mean larval development time at 8 µg/L was significantly longer than at 0 µg/L (mean difference = 7.84 days, *p* = 0.010). A similar trend was seen in the F2 larvae (mean difference = 8.47 days, *p* = 0.015). These delays were also evident in the pupal stage, particularly in the F1 group, where cadmium exposure at 8 µg/L significantly increased pupal development time compared to control (mean difference = 7.61 days, *p* = 0.014) (Figure 1).

**Figure 2:**
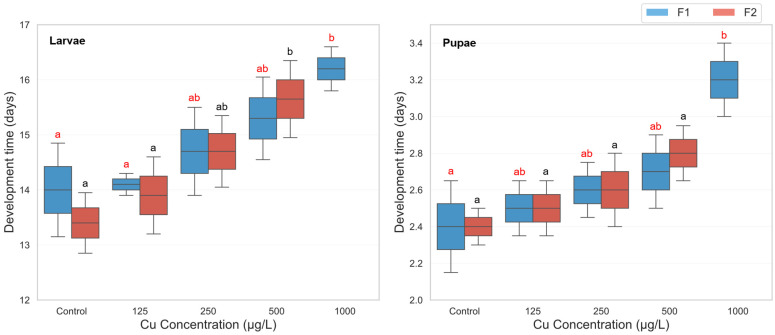
Larval and pupal development time in two successive generations of *Culex pipiens* following long-term exposure to varying concentrations of copper sulphate. Significant differences (*p* < 0.05, three-way ANOVA with Bonferroni test) in the developmental period are indicated by different red letters for the first generation and different black letters for the second generation.

The analysis revealed a highly significant main effect of copper sulfate concentration on development time (F(4, 39) = 14.97, *p* < 0.001, partial η^2^ = 0.606), demonstrating that increasing copper concentrations significantly delayed development. Similarly, a significant main effect of developmental phase was observed (F(1, 39) = 217.91, *p* < 0.001, partial η^2^ = 0.848), with larvae consistently requiring more time to develop than pupae. Additionally, the generation effect was also significant (F(1, 39) = 33.73, *p* < 0.001, partial η^2^ = 0.464), indicating that developmental delays varied between F1 and F2 generations. Significant two-way interactions were found between concentration and generation (F(4, 39) = 33.87, *p* < 0.001, partial η^2^ = 0.776), suggesting that the effects of copper concentration differed across generations. A three-way interaction between concentration, phase, and generation was also significant (F(4, 39) = 3.60, *p* = 0.014, partial η^2^ = 0.270), indicating that the impact of copper exposure on development time varied across both developmental stages and generations. Descriptive statistics revealed a consistent trend of prolonged development with increasing copper concentrations in the F1 generation. Larval development time in F1 increased from 14.0 days (control) to 16.2 days at 1000 µg/L, while pupal development extended from 2.4 to 3.2 days. However, in the F2 generation, exposure to the highest concentration (1000 µg/L) resulted in complete developmental failure (0.0 days), indicating severe toxicity (Figure 2). Post hoc Bonferroni comparisons confirmed that in the F1 generation, both larval and pupal development times at 1000 µg/L were significantly longer than in the control group (*p* < 0.01). In the F2 generation, development time at 1000 µg/L was significantly lower than all other treatments due to complete developmental arrest, with significant differences observed against all lower concentrations (*p* < 0.001) (Figure 2).

### 3.3. Fecundity and Egg Hatchability

**Figure 3:**
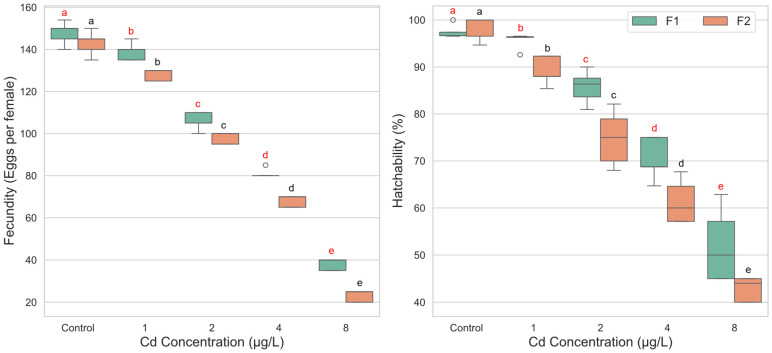
Fecundity and egg hatchability of *Culex pipiens* following exposure to varying concentrations of cadmium chloride across two successive generations. Boxplots labeled with different red letters denote statistically significant differences in fecundity and hatchability percentages among cadmium treatments within the first generation (two-way ANOVA followed by Bonferroni post hoc test, *p* < 0.05). Boxes marked with different black letters indicate significant differences within the second generation under the same statistical criteria.

The analysis of fecundity revealed a highly significant main effect of cadmium concentration (F(4, 39) = 189.802, *p* < 0.001, ηp^2^ = 0.951), indicating that cadmium exposure strongly influenced the number of eggs produced. As shown in the descriptive statistics, fecundity decreased in a clear dose-dependent manner, with mean eggs per female dropping from 145.40 in the control group to 30.50 in the 8 µg/L group (Figure 3). A significant main effect was also observed for generation (F(1, 39) = 31.777, *p* < 0.001, ηp^2^ = 0.449). Overall, the F1 generation (M = 102.36, SD = 41.03) exhibited significantly higher fecundity compared to the F2 generation (M = 92.00, SD = 44.06). However, the interaction between concentration and generation was not statistically significant (F(4, 39) = 1.205, *p* = 0.324, ηp^2^ = 0.110). This lack of a significant interaction suggests that the negative impact of increasing cadmium concentration on fecundity was consistent across both the F1 and F2 generations. Comparisons confirmed significant reductions in fecundity at all tested cadmium concentrations compared to control (*p* < 0.001). For both F1 and F2, each increase in concentration led to a significant decrement in fecundity (Figure 3).

For egg hatchability, the two-way ANOVA also showed significant main effects for both cadmium concentration (F(4, 40) = 178.383, *p* < 0.001, ηp^2^ = 0.947) and generation (F(1, 40) = 18.052, *p* < 0.001, ηp^2^ = 0.311). Critically, a significant interaction effect between concentration and generation was found (F(4, 40) = 3.676, *p* = 0.012, ηp^2^ = 0.269). This interaction indicates that the effect of cadmium concentration on egg hatchability differed between the F1 and F2 generations. In the F1 generation, a significant reduction in hatchability compared to the control (M = 97.46%) was observed starting at the 2 µg/L concentration (M = 85.71%, *p* < 0.001). The 1 µg/L concentration did not cause a statistically significant decline (*p* = 1.000). In contrast, the F2 generation demonstrated greater sensitivity. Egg hatchability was significantly reduced at all cadmium concentrations compared to its control group (M = 98.24%), including the lowest concentration of 1 µg/L (M = 89.20%, *p* < 0.001) (Figure 3).

**Figure 4:**
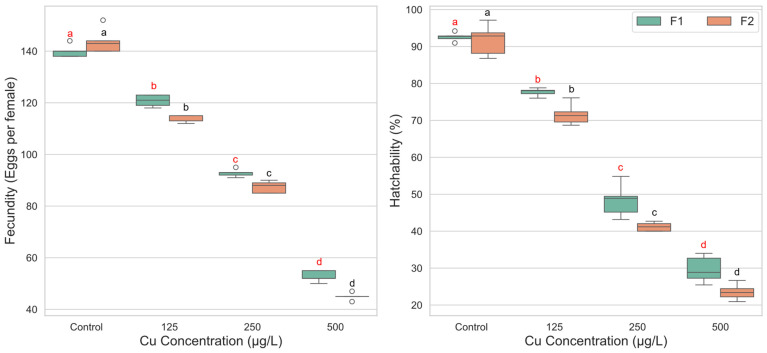
Fecundity and egg hatchability of *Culex pipiens* following exposure to varying concentrations of copper sulphate across two successive generations. Boxplots labeled with different red letters denote statistically significant differences in fecundity and hatchability percentages among copper treatments within the first generation (two-way ANOVA followed by Bonferroni post hoc test, *p* < 0.05). Boxes marked with different black letters indicate significant differences within the second generation under the same statistical criteria.

The two-way ANOVA revealed a significant main effect of copper concentration on fecundity (F(3, 31) = 194.144, *p* < 0.001, ηp^2^ = 0.949), indicating a strong, dose-dependent reduction in the number of eggs laid per female as exposure levels increased. A significant main effect for generation was also found (F(1, 31) = 13.723, *p* = 0.001, ηp^2^ = 0.307), with the F1 generation (M = 101.60) generally producing more eggs than the F2 generation (M = 97.55). Importantly, a significant interaction was detected between copper concentration and generation (F(3, 31) = 6.199, *p* = 0.002, ηp^2^ = 0.375). This interaction suggests that the magnitude of fecundity reduction caused by copper exposure differed between the two generations. Examination of the mean values shows that while the F2 generation exhibited slightly higher fecundity in the control group (M = 143.80) compared to the F1 control (M = 140.00), it experienced a more substantial decline upon exposure. For instance, the drop in fecundity from control to the 125 µg/L concentration was more pronounced in the F2 generation (a decrease of 29.8 eggs) than in the F1 generation (a decrease of 19.2 eggs), highlighting a greater sensitivity in the subsequent generation (Figure 4).

For egg hatchability, the two-way ANOVA identified a highly significant main effect of copper concentration (F(3, 32) = 694.663, *p* < 0.001, ηp^2^ = 0.985), with hatchability decreasing systematically as copper concentration increased. A significant main effect for generation was also observed (F(1, 32) = 18.757, *p* < 0.001, ηp^2^ = 0.370), with the F1 generation (M = 62.05%) showing consistently higher hatchability than the F2 generation (M = 57.01%). However, the interaction effect between concentration and generation was not statistically significant (F(3, 32) = 1.505, *p* = 0.232, ηp^2^ = 0.124). This indicates that the pattern of dose-dependent decline in hatchability was parallel for both generations. Hatchability was significantly lower at each increasing concentration level compared to the one before it (*p* < 0.001 for all comparisons) (Figure 4).

### 3.4. Histological Examination and Histomorphometry Measurements

**Figure 5:**
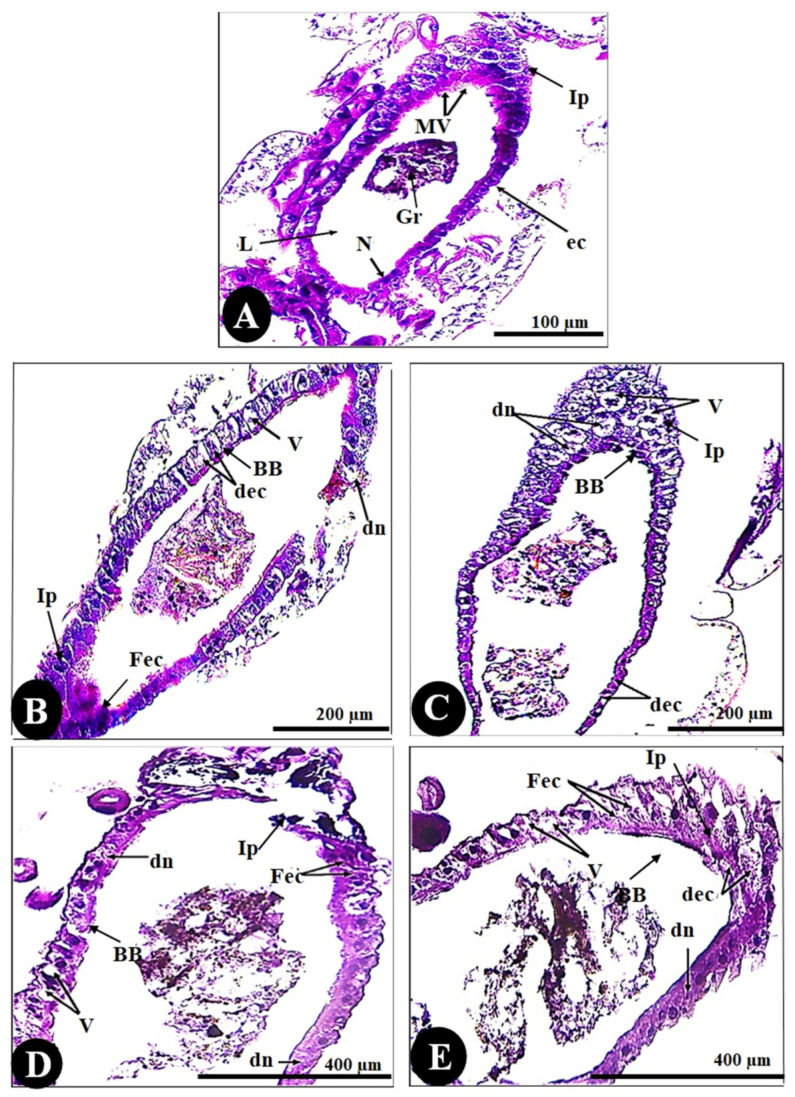
Cross-sections in the gut of the 3rd instar larvae of the mosquito *Culex pipiens*. (**A**) Control (X = 100), (**B**) larvae exposed to the LC_50_ concentration of CdCl_2_ in the 1st filial generation (X = 200), and (**C**) larvae exposed to the LC_50_ concentration of CdCl_2_ in the 2nd filial generation (X = 200). (**D**) Larvae exposed to the LC_50_ concentration of CuSo_4_ in the 1st filial generation (X = 400), and (**E**) larvae exposed to the LC_50_ concentration of CuSo_4_ in the 2nd filial generation (X = 400). (N) nucleus, (L) lumen, (Gr) granules, (BB) brushing border, (ec) epithelial cells, (Ip) inferior protuberance, (Mv) microvilli, (dec) degenerated epithelial cells, (dn) degenerated nuclei, (V) vacuolation, (Fec) fused epithelial cells.

As seen in the control sections (Figure 5), dark cells have normal nuclei, a well-developed brush border, normal sticky basement membranes, and normal intercellular connections over the entire lateral plasma membrane. All larvae that were chemically treated showed significant lesions that primarily affected the midgut epithelium and subsequently the caeca. Histopathological effects varied quantitatively based on treatment time and qualitatively based on where they occurred along the midgut. The anterior midgut protruded from the free surface in a nearly spherical shape. The other cell types were polygonal and appeared short; few epithelial cells had a brush border, whereas some had normal nuclei and a well-developed brush border.

**Figure 6:**
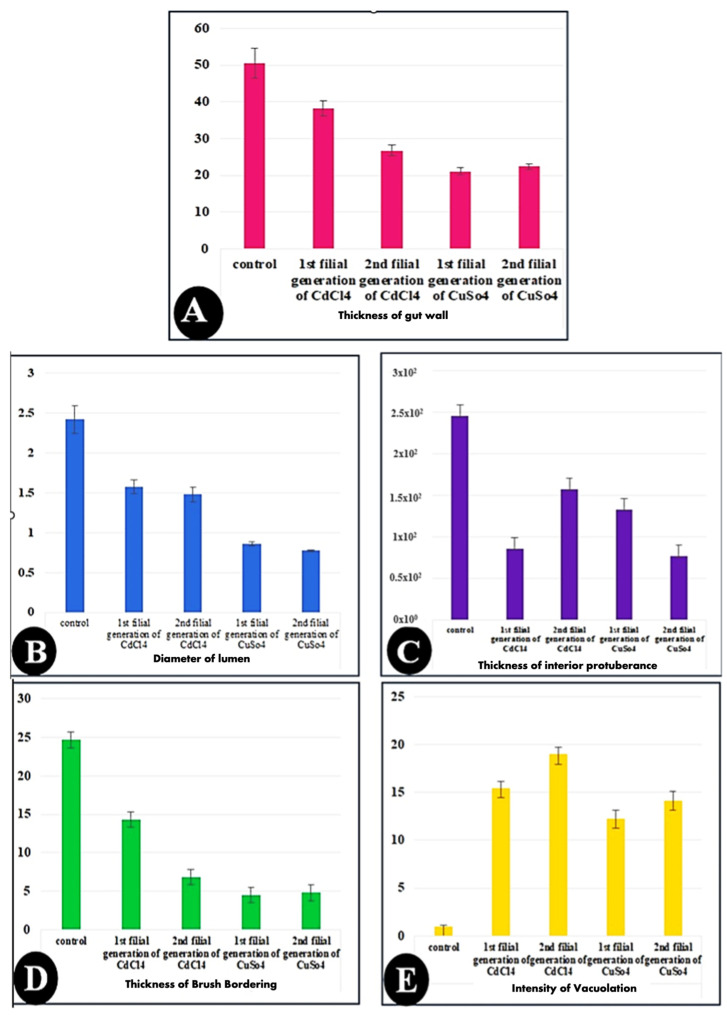
Bar charts showing the morphometric measurements of in the gut of the 3rd instar larvae of the mosquito *Culex pipiens*. (**A**) Control, (**B**) larvae exposed to the LC_50_ concentration of CdCl_2_ in the 1st filial generation, (**C**) larvae exposed to the LC_50_ concentration of CdCl_2_ in the 2nd filial generation, (**D**) larvae exposed to the LC_50_ concentration of CuSo_4_ in the 1st filial generation, and (**E**) larvae exposed to the LC_50_ concentration of CuSo_4_ in the 2nd filial generation. Values are represented as mean ± SEM, and n = 10 larvae.

Regarding histomorphometry measurements (Figure 6), the normal architecture of the gastric caecum of *Culex pipiens* larvae is composed of a single layer of cuboidal epithelial cells with a basal nucleus positioned on the basement membrane and a brush border of completely developed microvilli, measuring (24.66 ± 2.00 µm). The control gastric larvae recorded a wall thickness of (50.46 ± 4.11 µm). It is noticeable that the thickest observed area of the wall was the inferior protuberance of the caecum (245.71 ± 13.02 µm). The gastric caecum of *Culex pipiens* larvae exhibited a large lumen volume, which had granules of food particles (2.42 ± 0.17 mm). Out of all the cellular elements, the control larvae had fewer vacuoles (0.99 ± 0.14%).

On the other hand, larvae of *Culex pipiens* subjected to cadmium chloride showed significant differences in wall thickness (38.28 ± 2.05 µm) and brushing border (14.27 ± 0.71 µm) between the first and second filial generations. Table 3 shows that, in contrast to the first filial generation, the second filial generation of *Culex pipiens* larvae exposed to cadmium chloride showed a high level of significance in the thickness of the inferior protuberance (157.25 ± 4.11 µm) and the intensity of the vacuoles (18.96 ± 0.32%). However, there was no difference in the gut wall thickness, lumen width, brush border thickness, or vacuolation intensity between the two filial generations of larvae exposed to copper sulfate.

According to the preceding morphometric findings, copper sulfate enhanced cytotoxicity and generated greater cellular stress than cadmium chloride. This was clearly observed in the lumen diameter, brushing boundary, gut wall thickness, and inferior protuberance, as shown in Table 3.

## 4. Discussion

Heavy metal contamination is increasingly recognized as a major anthropogenic threat to freshwater ecosystems, with significant impacts on aquatic insects like *Culex pipiens*, which often breed in polluted environments, making them particularly vulnerable to the toxic effects of heavy metals [19]. Cadmium and copper are among the most toxic metals affecting *C. pipiens* larvae, according to research [20]. The present study provides strong evidence that sublethal exposure to cadmium chloride and copper sulfate can induce significant transgenerational effects on multiple biological traits, including development, reproduction, and gut morphology.

Larvicidal bioassays confirmed that both cadmium and copper exert dose-dependent toxicity, with cadmium chloride being more potent. Interestingly, first-generation larvae were more susceptible than the second, suggesting possible physiological acclimation or inherited tolerance. Such patterns are consistent with previous findings indicating increased resilience and even insecticide resistance in mosquitoes following prolonged environmental stress [7,21]. However, while generational differences were evident, the underlying mechanisms—whether genetic, epigenetic, or purely physiological—remain speculative in the absence of molecular validation.

The present study has shown that the delayed toxicity of heavy metals on the adult females resulted from larvae treated with the LC_50_ of the tested heavy metals significantly decreased the number of eggs. These results may be comparable with those obtained by El-Sheikh et al. [20] using different concentrations against the 3rd larval instar of *C. pipiens*. Developmental delays observed in the larval and pupal stages of both generations underscore the capacity of metal stress to disrupt key developmental processes. Prolonged development may affect not only population dynamics, but also ecological interactions and predator vulnerability. In the same context, Lee and Shin [22] reported that heavy metal contamination affected the size of a mosquito population by inhibiting the survival and growth of the mosquitoes.

Exposure of larvae to heavy metals can lead to a reduction in the fecundity of the resulting adult females. In this study, a reduction in fecundity of females resulted from larvae treated with the LC_50_ of the tested heavy metals was reported. This effect has been observed in various studies. For example, William et al. [23] demonstrated that *Chironomus riparius* laid fewer eggs in high cadmium concentrations (300 and 100 mg/L) than in lower concentrations or clean water. Additionally, the same findings were reported recently [6,24]. Reduced fecundity and hatchability in both generations further suggest that chronic metal exposure compromises reproductive fitness, likely through interference with endocrine signaling or oxidative damage to reproductive tissues [11,25,26,27,28].

Concerning the effect of heavy metals on reproduction, reports on the acute and chronic toxic effects of heavy metals on insect reproduction are frequent in the literature. Several studies have demonstrated pleiotropic chronic effects of Cd on insect physiology, affecting processes such as growth, development, reproduction, and/or hatchability [29,30,31,32]. However, the interruption of insect reproduction is an important and potent effect for heavy metals. Histopathological and histomorphometric analyses revealed marked midgut alterations, including epithelial disintegration, vacuolation, thinning of the gut wall, and loss of the brush border—particularly pronounced in copper-treated groups. These structural changes imply impaired nutrient absorption, weakened immune responses, and reduced metabolic efficiency, potentially compromising adult performance and vector competence. The cumulative nature of the damage across generations supports the hypothesis that chronic exposure may exert inherited effects, although further research is required to confirm epigenetic involvement.

From a vector management perspective, our results have important implications. Chronic exposure to low-level metal pollutants could alter vectorial capacity by modifying mosquito development rates, gut structure, and potentially susceptibility to pathogens. Given the ecological ubiquity of *Culex pipiens* and its role in disease transmission, such sublethal effects must be considered in integrated vector control strategies, especially in metal-contaminated environments. In summary, the integration of transgenerational biological assays with tissue-level analyses enhances our understanding of how environmental stressors shape vector biology. Future studies should employ molecular, genomic, and field-based approaches to elucidate the long-term ecological and epidemiological consequences of metal pollution.

Although this study provides novel insights, some limitations must be acknowledged. The absence of molecular assays—such as oxidative stress markers (e.g., SOD, CAT, MDA) or epigenetic regulators—limits our ability to conclusively identify mechanistic pathways. Additionally, all experiments were conducted under laboratory conditions; hence, field-based studies are necessary to validate these findings in natural habitats with complex ecological interactions.

## 5. Conclusions

This study provides robust evidence that sublethal exposure to cadmium chloride and copper sulfate induces significant transgenerational impairments in *Culex pipiens*, including delayed development, reduced fecundity, compromised egg viability, and severe midgut histopathological alterations. Cadmium exhibited greater larvicidal potency, while copper exposure caused more pronounced cellular damage. The observed generation-specific variations in sensitivity suggest possible physiological acclimation or inherited tolerance mechanisms, aligning with recent findings on transgenerational metal toxicity. These outcomes underscore the importance of considering chronic, low-level metal exposure in ecological risk assessments, particularly in vector ecology. The results also support the application of mosquitoes as sentinel organisms for aquatic heavy metal pollution monitoring. Future studies should integrate molecular and epigenetic analyses under both laboratory and field conditions to elucidate the mechanistic pathways of such transgenerational effects and assess their implications for vector competence and control strategies.

## Figures and Tables

**Table 1:** Larval and pupal toxicity of cadmium chloride against the mosquito *Culex pipiens* in two successive generations.

Generation	Instar/Stage	LC_50_ (LCL–UCL) (μg/L)	LC_90_ (LCL–UCL) (μg/L)	Regression Equation	χ^2^ (*d. f.*)	Statistic Summary
F1	1st larval instar	8.661 (6.82–12.35)	51.92 (29.82–128.25)	Y = 6.0X + 1.52	1.85 n.s. (4)	*p* < 0.001, R^2^ = 0.97, F = 131.836
	2nd larval instar	10.08 (6.99–19.38)	77.57 (33.94–384.35)	Y = 5.313X + 3.06	2.23 n.s. (4)	*p* < 0.005, R^2^ = 0.936, F = 59.069
	3rd larval instar	13.081 (7.8–40.5)	104.45 (35.49–281.82)	Y = 5.093X − 0.953	7.675 n.s. (4)	*p* < 0.002, R^2^ = 0.964, F = 107.268
	4th larval instar	20.8 (8.77–128.65)	293.43 (48.76–1266.8)	Y = 4.709X + 1.439	6.772 n.s. (4)	*p* < 0.001, R^2^ = 0.971, F = 135.188
	Pupae	23.62 (7.73–172.35)	263.88 (31.21–1152.41)	Y = 6.145X − 2.613	7.29 n.s. (4)	*p* < 0.004, R^2^ = 0.941, F = 64.829
F2	1st larval instar	6.84 (5.47–9.42)	48.4 (27.63–120.98)	Y = 6.78X + 3.98	3.519 n.s. (4)	*p* < 0.001, R^2^ = 0.974, F = 153.433
	2nd larval instar	9.165 (6.21–19.35)	55.66 (24.38–308.41)	Y = 6.565X + 0.087	12.71 n.s. (4)	*p* < 0.000, R^2^ = 0.994, F = 640.62
	3rd larval instar	12.76 (8.31–29.26)	88.25 (35.93–558.78)	Y = 4.895X + 0.564	3.825 n.s. (4)	*p* < 0.001, R^2^ = 0.981, F = 207.816
	4th larval instar	18.85 (7.89–94.06)	265.50 (152.43–940.43)	Y = 5.166X + 1.817	6.992 n.s. (4)	*p* < 0.001, R^2^ = 0.98, F = 201.141
	Pupae	44.91 (10.97–186.34)	285.383 (45.97–1076.22)	Y = 1.988X + 1.483	14.719 n.s. (4)	*p* < 0.002, R^2^ = 0.96, F = 97.078

(F1) first filial generation, (F2) second filial generation, (LC_50_) concentration that kills 50% of treated organisms, (LC_90_) concentration that kills 90% of treated organisms, (LCL) lower confidence limit, (UCL) upper confidence limit, (d. f.) degree of freedom, (χ^2^) Chi-squared, (n.s.) non-significant at (alpha = 0.05). Five replicates were used in each treatment. No mortality was recorded in the control group.

**Table 2:** Larval and pupal toxicity of copper sulphate (CuSo_4_) against the mosquito *Culex pipiens* in two successive generations.

Generation	Instar/Stage	LC_50_ (LCL–UCL) (μg/L)	LC_90_ (LCL–UCL) (μg/L)	Regression Equation	χ^2^ (*d. f.*)	Statistic Summary
F1	1st larval instar	175.63 (106.85–254.89)	1343.84 (634.62–8792.73)	Y = 0.064X + 27.04	9.522 n.s. (4)	*p* < 0.118, R^2^ = 0.483, F = 4.732
	2nd larval instar	219.94 (169.94–357.59)	579.74 (356.89–2607.64)	Y = 0.055X + 23.34	15.568 n.s. (4)	*p* < 0.202, R^2^ = 0.292, F = 2.65
	3rd larval instar	278.45 (198.89–423.28)	3911.48 (1512.89–8645.62)	Y = 0.047X + 24.061	8.429 n.s. (4)	*p* < 0.191, R^2^ = 0.315, F = 2.838
	4th larval instar	345.58 (211.33–4081.75)	1439.21 (494.44–4046.33)	Y = 0.119X + 4.176	13.302 n.s. (4)	*p* < 0.018, R^2^ = 0.947, F = 55.132
	Pupae	374.69 (328.44–428.76)	1514.50 (1178.57–2142.42)	Y = 0.082X + 7.960	6.079 n.s. (3)	*p* < 0.005, R^2^ = 0.928, F = 52.397
F2	1st larval instar	139.22 (92.047–182.74)	288.74 (207.79–1507.51)	Y = 0.191X + 11.086	9.875 n.s. (3)	*p* < 0.03, R^2^ = 0.91, F = 31.381
	2nd larval instar	202.53 (139.28–1004.29)	850.83 (383.71–3026.42)	Y = 0.194X + 4.46	2.939 n.s. (3)	*p* < 0.006, R^2^ = 0.983, F = 179.706
	3rd larval instar	220.52 (149.31–553.68)	1741.79 (631.05–6442.34)	Y = 0.161X + 7.84	5.173 n.s. (3)	*p* < 0.023, R^2^ = 0.931, F = 41.289
	4th larval instar	302.56 (236.76–468.69)	1816.3 (893.62–12,196.56)	Y = 0.133X + 5.396	4.670 n.s. (3)	*p* < 0.015, R^2^ = 0.955, F = 65.157
	Pupae	328.68 (280.97–400.18)	1459.71 (976.294–2930.32)	Y = 0.130X + 2.720	5.293 n.s. (3)	*p* < 0. 006, R^2^ = 0.983, F = 176.759

See footnote of Table 1.

**Table 3:** Comparison between the morphometric measurements (mean ± SEM) of *Culex pipiens* larvae exposed to copper sulfate and cadmium chloride on a range of life history traits throughout multiple generations (10 larvae per group). The measurements not sharing common superscripts denote significant differences (*p* < 0.05).

	Parameter	Thickness of Gut Wall (µm)	Diameter of Lumen (mm)	Thickness of Inferior Protuberance (µm)	Thickness of Brushing Border (µm)	Intensity of Vacuoles
Groups						
A		50.46 ± 4.11 ^c^	2.42 ± 0.17 ^c^	245.71 ± 13.02 ^c^	24.66 ± 2.001 ^c^	0.99 ± 0.14 ^a^
B		38.28 ± 2.05 ^b^	1.57 ± 0.08 ^a^	86.07 ± 3.53 ^a^	14.27 ± 0.71 ^b^	15.48 ± 0.67 ^c^
C		26.78 ± 1.57 ^b^	1.48 ± 0.08 ^b^	157.25 ± 4.11 ^b^	6.85 ± 0.40 ^a^	18.96 ± 0.71 ^d^
D		21.07 ± 1.02 ^a^	0.86 ± 0.02 ^b^	132.94 ± 6.52 ^b^	4.52 ± 0.17 ^a^	12.25 ± 0.90 ^b^
E		22.47 ± 0.73 ^a^	0.77 ± 0.011 ^a^	76.81 ± 2.20 ^a^	4.77 ± 0.45 ^a^	14.16 ± 0.92 ^b,c^
*p* value		<0.001	<0.001	<0.001	<0.001	<0.001

## Data Availability

The datasets used and/or analyzed during the current study are available from the corresponding author on reasonable request.
